# Address of J. W. Spaulding, M. D., before the Knox Co. Medical Society, July, 1st 1854

**Published:** 1854-09

**Authors:** 


ARTICLE IV.—*Address of* J. W. Spaulding, M. D., before the Knox Co. Medical Society, July, 1st 1854.

*Gentlemen of the Knox Co. Med. Society.*—In laying down the honors and retiring from the office of presiding over you for the last year, conferred upon me by your confidence and partiality, I feel it due to you, to return you my sincere thanks, and to establish a precedent which I hope will be followed by my successors, to the benefit of Medical science, and the establishment of professional union and courtesy. Gentlemen, we find a maxim laid down in a good book, upon which we may well ponder, it is this, "A house divided against itself cannot stand." We have adopted a code of ethics which if duly observed will carry us clear of all professional jealousy, bickering and strife, which sometimes unfortunately occurs among professional brethren, to their own discredit, and compromises the influence and interests of the profession.—The science of medicine is a noble science, and if rightly appreciated and understood by its votaries, will make them kind, generous and humane. It is a progressive science, and he who wishes to be the greatest benefactor to the human family, in arresting disease and turning aside her shafts, must drink *deep* and *often* at her fountains. And gentlemen, let me advise you to strive and contend, not for practice and dimes, but for knowledge and wisdom. Let us for a moment compare the science of medicine with some of the special systems of empiricism and charlatanry in our land. Homœopathy, claims to be an exact science, and Hannemann, like a philosopher of old, was enabled to exclaim *Eureka.* There are but two points in the so called science viz.: "Similia, Similibus Curanter" or, like cures like, and infinitesmal doses. We will look at the first point and see whether it holds good as a general law Hannemann says, that he utterly failed to cure disease, whenever he gave medicine in any considerable quantity, furthermore that it greatly aggravated the disease, and he only succeded when he used the minutest doses, the decillionth part of a grain or the thirtieth dilution. Now a person with one spark of common sense is enabled to see, that when a remedy in an inflammation is withheld, that is calculated to produce that state of the system, and a dose occasionally given of the thirtieth attenuation or dilution, which is inert, that the case is left to the efforts of nature, good nursing, and restricted diet to get well. There is a vast difference between recoveries and cures, that is sometimes not appreciated, and when nature restores one case that is doubtful or considered so under an empiric it is considered marvelous, and heralded over the land, whereas the regular practice may cure forty desperate cases, and it is no more than they expect of us. These things, after all, satisfy me that the community consider it a wonderful escape, and we think the Homœopathists are confuted by their own argument.

We will next notice the infinitesmal doses, the thirtieth dilution which Hahnemann declares is infallibly the most appropriate dose, as well for chronic as for acute diseases, consists of a decillionth of a grain or of a drop of the drug, thus we have 1,000,000,000,000, 000,000,000,000,000,000,000,000,000,000,000,000 000 000 000, of a grain, or drop.

The Homœopathic list of drugs, includes a number of medicines that possess (according to Homœopathy) the power of producing various moral and religious states and symptoms. Sulphur produces in a healthy person, the feeling of "despair of eternal salvation." A dose of pulsatilla, produces, "despair of eternal happiness with continued praying, hymns, and devout aspect." Veratrum produces "extraordinary taciturnity, with oaths on the slightest provocation and raving about religious matters." It is the doctrine of Hahnemann that the most of chronic diseases are produced by psora or itch, and Dr. Muir has discovered a new and grand specific, which consists of the human louse, or "pediculias capitis" Dr. Muir found that doses of louse tea were capable of creating 283 different symptoms to which he devotes twelve pages to show it to be a marvelous specific for the itch. Dr. Herring recommends swallowing "bugs in the thirtieth dilution" for curing the inflammation arising from bug bites. You will find contrasted with the above in the regular science, its votaries investigating in every department, and whenever a substance is found, and proved by experiment and investigation, calculated to alleviate human suffering and misery, it is received as a boon to the race, whether coming from the animal, vegetable or mineral kingdom, you also find powerful minds investigating every department of the science and bringing in their knowledge to the general stock. But, gentlemen there are other empirics in our land, to wit.: Hydropathy and Botanic. Cold water, or rather water at various temperature is a therapeutic agent of great value, and has been in the hand of the profession for centuries, however, the idea embraced by some that water is a specific for all the ill that flesh is heir to, to the exclusion of all else that a bountiful providence has provided, is evidence of an ignorant and contracted mind, and as well might the mariner when he leaves port place the bow of his ship towards the desired haven, lash the helm, throw overboard the charts, compass and quadrant. and notwithstanding the variable winds and currents, expect to reach his port in safety, without being driven from his direct course, or buffetted by the winds and waves. The botanic comes next and is the most contemptible of all. "Thompson and all his crew," they deal in vegetable remedies because they are *harmless* and exclude the *poison minerals*, and endeavor by pampering the prejudices of the credulous to succeed, not by science, learning and skill, but by loud boastings and pretensions.

There are many honest persons that have been made to believe that botanical remedies are harmless, and yet effectual; also, that there is some secret that the regular profession have not discovered; they, honest souls, don't know that one drop of hydrocanic acid—a vegetable acid—will produce death in an adult in two minutes, and that strychnia will produce death in ten minutes; and if they could read the journals, and see the number of deaths from Lobelia poisoning, they might think it required some skill and judgment in the dispensation of botanical remedies. Another idea—I have frequently seen pasted hand-bills by the above gentry, making boastful pretensions, and repudiating all "mineral remedies," and covertly make use of more mercury than any regular practitioner; and if ptyalism followed, it was the old calomel they were expelling from the system. But many, from inattention, or want of comprehension, fail to see that in medicine knowledge is power, and ask for the practical evidence of our ability to treat disease. And here we shall be able to make our strongest claim upon public confidence, and our most invulnerable defence against the attacks of our enemies; but in casting around for a point at which to commence, so innumerable are the triumphs of our art, that their very abundance creates embarrassment, as neither mountain nor ocean impose a successful barrier to our onward progress, so neither the rapidly ebbing currents of life through the several arteries, nor the terrific throes of obstructed labor defy our skill, for we quietly pass a ligature around the vessel in one case, and, by the application of a safe and efficient instrument, give prompt relief to almost superhuman suffering, and save two lives, in the other; we cut short, in a few days, the miasmatic fevers of the great West, which, without our aid, prove fatal, or run a course of weeks, or even months. By vaccination we guard the community from a dangerous and loathsome disease.

But, notwithstanding our claims to the confidence of the community, there is a class who are excessively credulous, and who are on the look-out for some new way, and as soon as a new system is promulgated, follow immediately after, shouting praises to the progressive spirit of the age. They will also take great pains to patronise and recommend a boastful pretender, if he gives them a history of some marvelous cures that he has performed after all the doctors had failed.

The above class it is useless to try to convert to sound principles; it would be like casting pearls before swine, and we had better let them alone; but, notwithstanding all the detraction and abuse, the true science will prevail. But I must refrain from pursuing the subject further, and close by hoping that this society will be united in close ties of courtesy and kindness, and continue the search after truth, until we shall become a beacon that will be seen and known afar.

Again, gentlemen, accept my thanks for your kindness and confidence, and rely on my best endeavors in co-operating with you to sustain the interest and honor of the Knox County Medical Society.

